# Building a collaborative ecosystem across the IDeA-CTR networks in response to a public health emergency

**DOI:** 10.1017/cts.2025.10098

**Published:** 2025-07-16

**Authors:** A. Jerrod Anzalone, Sharon Patrick, Amber Abel, Brad Price, Elizabeth Reisher, Kent Ripplinger, Mary Emmett, Ronald Horswell, San Chu, William B. Hillegass, Francisco S. Sy, Brian Melancon, H. Timothy Bunnell, Lucio Miele, Mary Helen Mays, Joseph Keawe‘aimoku Kaholokula, Elizabeth S. Chen, Karen M. Crowley, Indra Neil Sarkar, Susan L. Santangelo, Clifford J. Rosen, Jeremy Harper, David Bard, William Beasley, Sally L. Hodder

**Affiliations:** 1 University of Nebraska Medical Center, Great Plains IDeA-CTR, Omaha, NE, USA; 2 West Virginia University, West Virginia Clinical and Translational Science Institute, Morgantown, WV, USA; 3 University of North Dakota, Translational Science Engaging North Dakota Program, Grand Forks, ND, USA; 4 CAMC Health System, West Virginia Clinical and Translational Science Institute, Charleston, WV, USA; 5 Pennington Biomedical Research Center, Louisiana Clinical and Translational Science Center, Baton Rouge, LA, USA; 6 University of Mississippi Medical Center, Mississippi Center for Clinical and Translational Research, Jackson, MS, USA; 7 University of Nevada Las Vegas, Mountain West Clinical and Translational Research Infrastructure Network, Las Vegas, NV, USA; 8 Nemours Children’s Health, ACCEL – Delaware Center for Translational Research, Wilmington, DE, USA; 9 Louisiana State University Health Sciences Center, Louisiana Clinical and Translational Science Center, New Orleans, LA, USA; 10 University of Puerto Rico, the Hispanic Alliance for Clinical and Hispanic Research, San Juan, PR, USA; 11 University of Hawai‘i at Mānoa, Center for Pacific Innovations, Knowledge and Opportunities, Honolulu, HI, USA; 12 Brown University, Advance Rhode Island Clinical and Translational Research, Providence, RI, USA; 13 Tufts University School of Medicine, MaineHealth Institute for Research, Northern New England Clinical and Translational Research Network, Scarborough, ME, USA; 14 Owl Health Works, LLC, Indianapolis, IN, USA; 15 University of Oklahoma Health Sciences, Oklahoma Shared Clinical and Translational Resources, Oklahoma City, OK, USA

**Keywords:** IDeA-CTR, team science, collaborative analytics, national COVID cohort collaborative (N3C), COVID-19, socio-technical ecosystem

## Abstract

**Introduction:**

The urgency and scale of the COVID-19 pandemic demanded a coordinated response from public health agencies and the biomedical research community. The National COVID Cohort Collaborative (N3C) was established as a centralized enclave in 2020 to support the study of COVID-19 across the U.S. The Institutional Development Award for Clinical and Translational Research (IDeA-CTR) centers enhanced N3C’s national response by bringing representation from rural and medically underserved communities. This improved the representation of our diverse populations in the N3C Enclave and its use for research by IDeA-state investigators.

**Methods:**

We developed an organizational structure across the IDeA-CTRs to improve research productivity in resource-challenged areas of the U.S. This socio-technical ecosystem, informed by community input, included a governance committee and two workstreams. The operations workstream focused on data management and regulatory compliance, while the navigation, education, analysis, and training (NEAT) workstream supported educational and analytical activities for the N3C Enclave.

**Results:**

Our collaborative approach led to participation by 12 IDeA-CTRs, representing over 400 investigators from 23 sites. The shared governance, investigator engagement, and resource pooling enhanced research productivity and engagement with researchers across IDeA states. Participation in this IDeA-CTR N3C consortium enhanced informatics research capacity and collaboration across the IDeA-CTRs for participating networks.

**Conclusions:**

This collaborative model provides a roadmap and framework for future efforts among IDeA-CTRs and other academic partnerships. The socio-technical ecosystem fostered collectivism and team science, enabling the consortium to achieve far more than isolated efforts could, offering valuable insights for interdisciplinary research across geographically dispersed communities.

## Introduction

The COVID-19 pandemic altered the course of history in many ways, including the loss of more than 1.2 million U.S. lives [1], highlighting the need for a cross-disciplinary, coordinated response. The National COVID Cohort Collaborative (N3C), a next-generation registry [2] established by the National Center for Advancing Translational Sciences (NCATS) and the Center for Data to Health (CD2H) in mid-2020 [3], was developed to address this urgent need. N3C enables individuals and teams to collaboratively engage in the study of COVID-19 and related sequelae, codifying open governance practices [3], and community guidelines [4] that embed Findability, Accessibility, Interoperability, and Reusability (FAIR) [5] guiding principles into the infrastructure to support transparency and reproducibility in the public health response.

Extensive effort has gone into N3C’s governance structure [3,6], data quality feedback cycle [7], linkage with other NIH-managed efforts [8,9], and commitment to team science [10], as documented elsewhere. N3C’s community has organized into clinical and cross-cutting domain teams [11] to address specific research areas, while a comprehensive governance structure ensures data security, ethical access, and equitable attribution to support widespread collaboration and rapid, impactful analysis [6]. To date, N3C represents the largest limited data set gathered for COVID-19 research in the U.S. [12], providing a valuable resource for studying the pandemic’s diverse effects, including systemic health disparities. Despite the comprehensive efforts supporting N3C, the pandemic’s disproportionate impact on rural [13,14] and medically underserved [15,16] populations underscored the need for targeted contributions to address health disparities in these vulnerable communities [17,18].

The Institutional Development Award for Clinical and Translational Research (IDeA-CTR) program was established in 2012 by the National Institute of General Medical Sciences (NIGMS) to support research infrastructure and workforce development, focusing on the most pressing health conditions in 23 states and Puerto Rico that have historically had lower rates of NIH funding [19]. With their strategic presence serving multiple underrepresented and underserved communities across the U.S., IDeA-CTRs are uniquely positioned to address health disparities facing the communities they serve.

**Figure 1.**
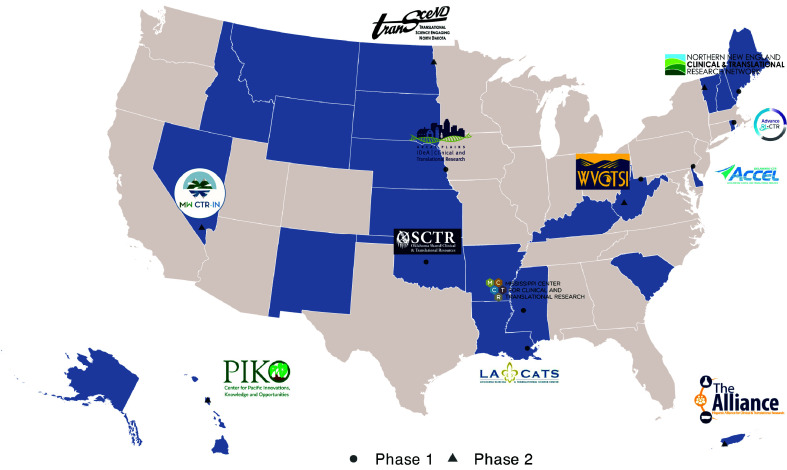
IDeA-CTR networks participating in N3C, 2020-2023. Includes the IDeA-CTR network sites participating in N3C in phases 1 (circle) and 2 (triangle). IDeA states are highlighted in **blue**. 14 total IDeA-CTR-associated sites participated in the network from 12 IDeA-CTR networks.

Through joint participation in N3C, the IDeA-CTR N3C consortium was established early in the pandemic. This resulted in significant clinical data contributions from these communities, enabling a better understanding of the pandemic’s impacts on vulnerable and resource-poor populations. The consortium also allows better representation of research interests that impact IDeA states and their populations. Beginning with an initial pool of eight funded sites in 2020, this consortium grew to include 14 sites from 12 IDeA-CTR networks in 2023 (Figure 1). The IDeA-CTR’s involvement in N3C complemented existing N3C initiatives while catering to the specific needs of investigators from IDeA states and their participation criteria. Our efforts should be viewed as complementary to existing N3C processes, extending the reach and utility of N3C through tailored support for a more diverse research community.

In response to the diverse research needs of IDeA states and their investigators, we established a socio-technical collaborative ecosystem that included cross-IDeA-CTR investigator engagement, a centralized navigation team for onboarding new members, and resource pooling to support use of the N3C Enclave. We present a scalable framework and roadmap, which features a governance committee and two primary workstreams focused on operations and educational and analytical initiatives, which extends the existing N3C ecosystem [6] and the requirements for shared infrastructure across research organizations [20]. Although individual clinical research networks have developed governance structures for participation [21–24], none specify requirements for collaboration in a centralized resource for broad research access. While N3C governance offered broad, consortium-wide support, our approach focused on centralized resource sharing and site-level mentorship to ensure consistent engagement across IDeA institutions and promote N3C’s use in local research communities. Our framework addresses this gap, promotes collaboration, ensures equitable representation, and enhances research capacity in multi-institutional collaboratives. While operating within the requirements for site participation in N3C, the framework preserved the autonomy and research priorities of IDeA-CTR investigators.

## Site requirements for n3c participation

Through October 2024, N3C includes data from 85 U.S. health systems, representing over 23 million patients. While this number indicates broad participation in the N3C Enclave, initial site participation in N3C involved a significant investment in data management and institutional commitment to transform electronic health record (EHR) data into a research-ready format. The IDeA-CTR N3C consortium prioritized several requirements early, categorized under two primary areas: data transmission and data utilization. These requirements encompass the technical aspects of preparing and submitting data and the organizational and methodological preparedness for leveraging the N3C Enclave for research.

## Data transmission requirements

Participation in N3C as a data-contributing site requires significant efforts to establish or maintain an enterprise data warehouse for research (EDW4R) [25]. This entails transforming clinical data into structured, interoperable formats for research use [26]. Unlike NCATS-funded Clinical and Translational Science Award (CTSA) hubs [27], the other major N3C data-contributing partners, IDeA-CTRs lack mandatory research informatics cores and are not obligated to maintain an EDW4R [28]. Several studies [29–32] have explored the informatics requirements of CTSA hubs; however, limited efforts have addressed the specific needs of IDeA-CTRs in participating in network research. IDeA-CTRs participating in N3C face varying levels of readiness due to the high demands of creating an EDW4R, a requirement for participation in network research. Sites contributing to N3C undertake several foundational activities to ensure successful participation (Table 1). Organizations without an EDW4R often lack the expertise to leverage big data resources.

**Table 1.** Steps required to establish and maintain an enterprise data warehouse for research

Step	Description
Data Accrual	Collecting health-related data from various sources within the healthcare system.
Standardization	Aligning data with standardized terminologies and formats according to a common data model (CDM).
Governance and Privacy	Establishing frameworks to address privacy, consent, and ethical considerations in compliance with regulations such as HIPAA.
Technology Infrastructure	Enhancing IT infrastructure to support large-scale data storage, processing, and analysis.
Participation and Collaboration	Sharing de-identified data, contributing to multi-center studies, and engaging in network-wide initiatives.
Quality Assurance and Improvement	Continuously monitoring and improving data quality and completeness to ensure the reliability of research findings.

The initial phase of EDW4R adoption includes data accrual from diverse healthcare sources such as EHRs, billing systems, and patient registries, requiring collaboration with IT and clinical staff to accurately map complex data into research-ready formats [33]. Following accrual, data undergo transformation into a research common data model (CDM) supported by N3C (OMOP [34], PCORnet [35], ACT [36], or TriNetX [37]). Standardization promotes interoperability while fostering research collaborations across healthcare systems by having common metrics and terminologies. Establishing EDW4R requires governance frameworks ensuring HIPAA compliance and data privacy, alongside secure, scalable research informatics infrastructure to support large-scale storage, processing, and analysis of diverse data sources while protecting patient privacy and enabling research.

N3C participation involves further requirements, including frequent data transmissions (often weekly) with updates to critical elements such as SARS-CoV-2 testing, viral variant sequences submitted to GenBank [38] linked to N3C submissions, and evolving terminologies on treatments and vaccines. Unlike the federated query approach typical in the U.S., centralized data sharing requires enhanced technological and governance capacities to transmit data to a single environment securely [7].

## Data utilization requirements

Using real-world data (RWD) within research networks like N3C is complex. RWD originates from clinical care and billing rather than research, necessitating expertise in biostatistics and informatics for accurate analysis [39]. Research teams must handle high-dimensional data and recognize EHR limitations, requiring a nuanced understanding of data quality issues like irregular sampling, missing data, and temporal or geographic variability [40].

Researchers must address data heterogeneity and generalizability issues from the study design phase to ensure research validity. Variations in documentation and data mapping across healthcare systems can introduce biases, complicating EHR data analysis and interpretation [41,42]. To manage these challenges, standardization and data harmonization techniques are essential for improving EHR-based study reliability [43].

While IDeA-CTRs [44] possess expertise in clinical informatics and data analysis, real-world data science capacity remains a common challenge. Data science expertise is needed to effectively analyze RWD gleaned from different non-integrated datasets. Additionally, researchers must navigate complex governance, privacy, and ethical standards to uphold patient trust and research integrity [45]. These regulatory considerations become especially critical during public health emergencies when research findings face heightened public scrutiny.

## The evolution of a socio-technical ecosystem to navigate pandemic challenges wwithin the idea-ctr n3c consortium: challenges in data contribution, data utilization, and collaborative research

The IDeA-CTR N3C consortium faced challenges related to data contribution, data utilization, and collaborative research during the COVID-19 pandemic. These were navigated by developing a socio-technical ecosystem that fostered innovation and collaboration across the consortium. Multidisciplinary collaboration among clinical experts, biologists, informaticians, biostatisticians, and data scientists added significant value at the institutional and consortium levels. This ecosystem–characterized by shared governance, education, navigational project support, and a shared pool of analysts and project managers–has emerged as a framework for future interdisciplinary endeavors that IDeA-CTR collaborative efforts may undertake beyond N3C.

To address these challenges, the consortium adopted a modular, smaller-scale sub-network within the broader N3C ecosystem, similar in concept to agile “two-pizza” teams (i.e., cap group size to a count easily fed by two pizzas), popularized by Amazon. This structure enabled tighter collaboration, reduced communication overhead, and greater ownership among participants, factors shown to enhance team productivity and responsiveness in complex systems [46]. By operating as a cohesive, high-trust collective, the consortium could move more nimbly than larger N3C workstreams while still aligning with shared objectives and infrastructure. This structure was supported by Conway’s Law [47], which asserts that the architecture of complex systems mirrors the communication structures of their creators. With N3C servicing over 80 sites, at varying levels of informatics maturity, a smaller, tightly networked IDeA-CTR subgroup enabled communication patterns and governance structures that more closely matched its institutional capacity and needs.

While N3C provides a mature, transparent governance ecosystem and working groups for shared analytics and data governance (e.g., DUAs, DURs, codes of conduct), the IDeA-CTR consortium added an intermediary scaffolding layer with unique functions:

1. Tailored Onboarding & Capacity-Building:Unlike N3C’s broadly scoped support, our consortium provided customized training, analytical templates, and readiness assessments aligned with the baseline capacity and priorities of IDeA state institutions, which often lacked clinical research informatics infrastructure before the pandemic.
2. Collectivized Project Management & Shared Resources:Borrowing from agile principles of distributed leadership, we deployed a centralized team of project managers and analysts across sites. This contrasts with N3C’s decentralized model, which enables consistent and efficient coordination, as well as reduced redundant effort.
3. Governance Calibration & Trust-Building:While N3C’s governance structures are robust, our smaller subgroup enabled more equitable decision-making and frequent alignment meetings (monthly dashboards, monthly workstream reviews) that fostered local trust and accountability, not always feasible in a much larger cohort.
4. Rapid Feedback Loops & Adaptive Learning:The IDeA-CTR model supported rapid iteration: sites could trial a data submission or template, share lessons, and collectively adjust processes more swiftly and with less overhead than would have been possible in full N3C channels.

**Figure 2.**
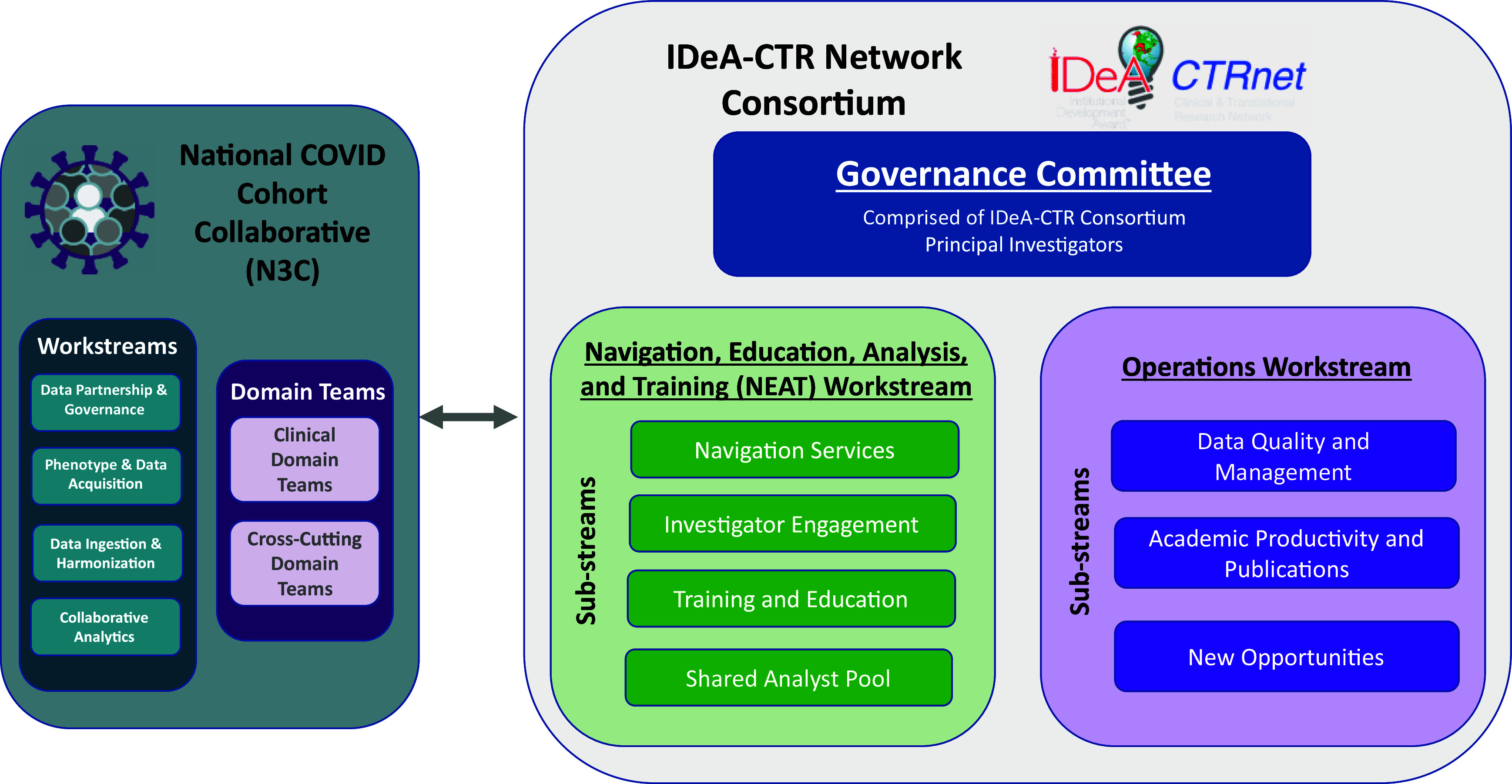
IDeA-CTR N3C network consortium organizational structure. Describes the organizational structure of the IDeA-CTR N3C consortium (right) and illustrates its relationship to the existing community and governance structures in N3C (left). Many IDeA-CTR investigators are participating in N3C workstreams and domain teams.

N3C now maintains a mature data governance structure and ecosystem [6], including several working groups dedicated to advancing community-driven, transparent decisions to support a learning health network [3] (i.e., a collaborative network to improve healthcare delivery and outcomes through the continuous cycle of learning and improvement) within the N3C community. The IDeA-CTRs joined N3C through a single funding mechanism in 2020, led by the West Virginia Clinical and Translational Sciences Institute (WVCTSI), which provided centralized support and project management to coordinate participating institutions. This governance model was designed to operate within and complement N3C’s existing structure, providing additional scaffolding to support IDeA-CTR institutions, many of which lack research informatics infrastructure or prior engagement with national data networks. The IDeA-CTR consortium expanded these efforts to address the specific needs of its institutions and investigators, while partnering with the broader N3C collaborative, as illustrated in Figure 2.

The IDeA-CTR consortium established a governance committee and two workstreams, operations and NEAT (navigation, education, analysis, and training), to support data transmission, usage, and collaborative research while aligning with IDeA-CTR site priorities. The consortium employed a collectivist model with centralized decision-making to reflect shared IDeA-state initiatives, fostering inclusivity, transparency, and trust. Shared project managers and centralized governance ensured collaborative and efficient decision-making, offering a scalable roadmap for future multi-institutional research based on our experience in N3C.

Maintaining a governance structure that incorporates participation from all consortium sites is a key feature, enabling shared decision-making and fostering a culture of inclusivity, transparency, and trust grounded in the needs of participating sites. The consortium’s monthly meetings maintained alignment with IDeA-CTR priorities and emerging challenges in data transmission and use within N3C, including the development of a dashboard to drive performance. The consortium’s organization evolved into two primary workstreams, operations and NEAT, that operate under the aegis of the governance committee. These two workstreams supported the data transmission and use requirements to create a learning community within the IDeA-CTRs.

## Operations workstream

The operations workstream supported initial and ongoing data transmissions and enhancements for data-contributing IDeA-CTR sites. This workstream was responsible for the readiness of sites to transmit data to N3C, which required executing legal contracts and data cleaning along with other activities as required by N3C. Additionally, the operations workstream hosted investigator engagement events open to all IDeA-CTR sites and encouraged IDeA-CTR teams to join N3C committees and domain teams.

The IDeA-CTR N3C operations meetings were important to the N3C initiative and its progress. These meetings discussed the required N3C process, engendering understanding among sites, and offered an open forum to discuss challenges and barriers to successful N3C participation. Given the various stages of familiarity with participation in research networks, the governance committee quickly realized the value of centralized milestone tracking across participating sites. Initial milestones included the completion of data transfer and use agreements, which were on file with NCATS. Individual IDeA-CTR sites had to work with local institutional review boards to ensure compliance documentation was vetted and approved. Three sub-streams emerged to support data quality and management, academic productivity and output, and new opportunities.

### Data quality and management sub-stream

The Data Quality and Management sub-stream played a critical role in preserving the integrity and utility of consortium-contributed data. This sub-stream supported site data preparation, mapping, and transformation from varied healthcare systems, ensuring uniformity within the N3CEnclave. The IDeA-CTRs represent various health data systems, source CDMs, and prior participation in network research before N3C. Among IDeA-CTRs, sites contributed data in different source CDMs, including five (45%) OMOP, two (18%) PCORnet, and four (36%) TriNetX. Sites also worked across different source health systems, with seven (64%) contributing data from a single health system, three (27%) from multiple health systems, and one (9.1%) from a health information exchange (HIE). Before N3C, seven (64%) sites participated in prior EHR-based research networks, including five (45%) participating in funded network studies.

Given the varying readiness for participation in N3C and differences in source CDMs, the data quality and management sub-stream worked with sites to expedite participation. By sharing best practices and identifying collective barriers, this sub-stream supported sites’ initial participation as data contributors in response to feedback requirements from the N3C ingestion and harmonization process, which included report cards for enhancing data quality [7]. This sub-stream supported executing data transfer agreements [48] and privacy-preserving record linkage [8] (PPRL) contracts, ensuring timely and secure data transfer into the N3C Enclave.

In addition to the basic requirement to contribute institutional data to N3C, the IDeA-CTR consortium made more ambitious attempts to support advanced data transmissions. The consortium prioritized data quality and completeness through quarterly assessments at the site level, complementing existing N3C site quality assessments [7]. Given the heterogeneity of data models and site maturity, formal standardized data quality metrics were not collected. Supplemental Table 1 summarizes the quarterly engagement and reporting metrics used to monitor consortium activities, while internal site reviews and peer vetting of analytic pipelines provided an additional layer of quality assurance, complementing N3C’s centralized evaluations. This sub-stream provided data mart managers a platform to exchange best practices and address issues as they occurred.

### Academic productivity and publications sub-stream

The Academic Productivity and Publications sub-stream emphasized disseminating the consortium’s research findings across the IDeA-CTRs, ensuring rigorous publication review processes, and fostering a culture of ongoing scientific dialogue among IDeA-CTR consortium members. The consortium shared scientific findings internally and externally with the broader scientific community. Internal sharing was achieved through presentations of research in progress at monthly IDeA-CTR-wide consortium meetings, where members discussed ongoing projects, preliminary findings, and methodologies. These forums complemented N3C-wide knowledge exchanges by promoting site-specific engagement with N3C tools and increasing research capacity among CTR investigators.

In addition to standard review processes, the consortium implemented an internal feedback process to guide project development and reduce duplication of similar efforts. Investigators received input at multiple stages, focusing on study design, cohort definitions, and analytic approaches. This feedback was guided by principles of scientific rigor, transparency, and alignment with IDeA-state research priorities. We found that early feedback reduced time to project completion, improved analytic consistency, and minimized overlap across studies. This iterative process complemented N3C’s centralized review, which emphasized attribution and data privacy.

### New opportunities sub-stream

The New Opportunities sub-stream focused on facilitating the incorporation of new data elements, including multimodal data, exploring funding opportunities, and identifying research avenues outside of N3C that align with the consortium’s interests. One example was prioritizing new data elements to support the evolution of SARS-CoV-2 research throughout the pandemic. This included several key data enhancements for data-contributing sites, which resulted in several members providing enhanced data beyond the EHR to provide a robust landscape to study COVID-19. This included linking emerging SARS-CoV-2 genetic variants with GenBank (six sites), incorporating de-identified clinical images into the N3C database (seven sites), and participating in PPRL (seven sites) to link patient records with claims data and externally validated death records [49]. These efforts aimed to enrich the data available for research, thereby enabling more comprehensive and nuanced analyses of COVID-19’s impacts.

The New Opportunities sub-stream also identified emergent funding opportunities that support the consortium’s research. By informing members of relevant funding mechanisms, the consortium could pursue its research objectives, including opportunities outside of N3C. This encouraged collaboration across the IDeA-CTRs, which resulted in several non-COVID-19 grant submissions and projects.

### Consortium support for regulatory navigation

To assist sites in meeting regulatory milestones, the consortium provided additional support beyond N3C’s centralized mechanisms. This included guidance for local IRB submissions, adaptations to institutional policies, and clarification of data use and transfer agreements (DUAs and DTAs). Shared templates and documentation streamlined approvals, and site navigators, along with a consortium-wide project manager, were available to assist sites in efficiently executing agreements. These efforts complemented N3C’s centralized support by addressing institutional variation and accelerating site readiness for participation.

## Navigation, education, analysis, and training (NEAT) workstream

**Figure 3.**
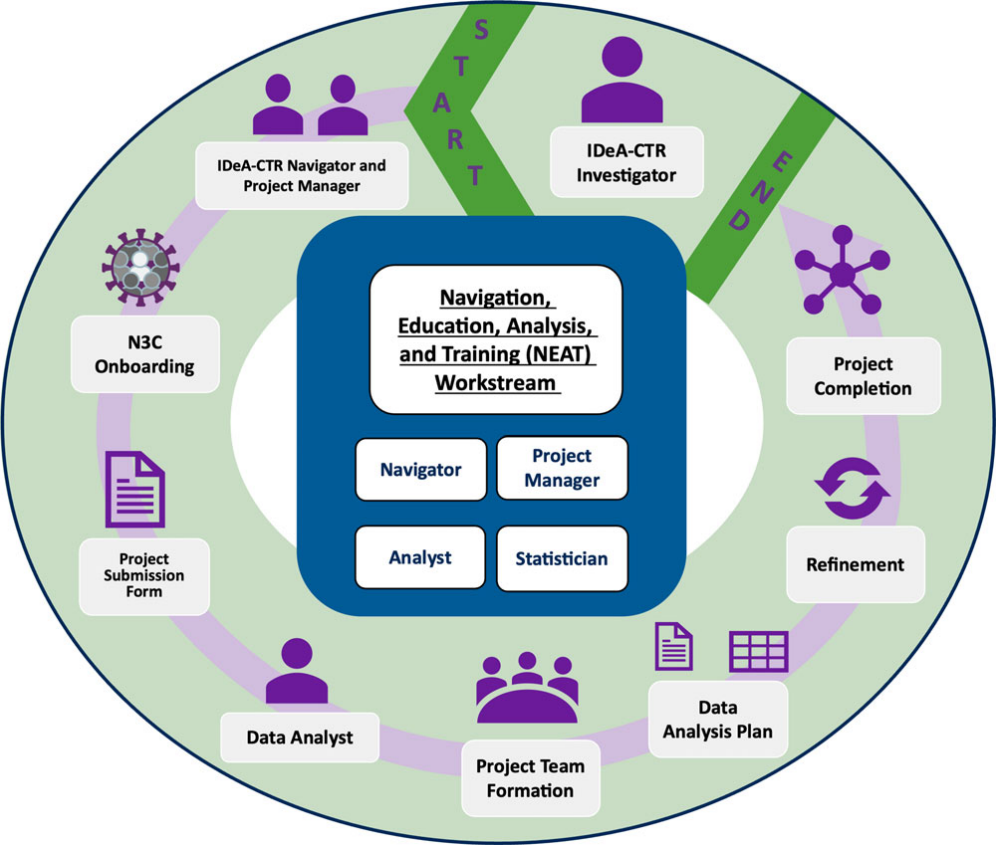
Investigator workflow through the NEAT workstream. Details the workflow through which an IDeA-CTR investigator initiates a project and how it is processed through the navigation, education, analysis, and training (NEAT) workstream through project completion. This process was initiated early in the IDeA-CTR N3C consortium formation to allow investigators from across organizations to pool resources and collaborate across the networks.

The NEAT workstream represented a novel approach to enhancing the consortium’s ability to effectively utilize the vast data collected and shared within the N3C Enclave. This workstream involved providing hands-on navigation, developing and delivering educational content, promoting rigorous data analysis, and offering comprehensive training to consortium members, including data science graduate students and medical trainees. The NEAT workstream involved an end-to-end workflow to help investigators rapidly transition from a research idea to a finished project (Figure 3), even in cases where investigators do not have access to data analysts or statisticians at their home institution able to work in N3C.

The NEAT workstream met biweekly to coordinate resources across the consortium, share code and analytic resources, and distribute new governance requirements. While this type of investigator-support workflow has been deployed at individual sites [30,50], its use throughout a multi-site consortium has been described less frequently in the literature. N3C supported participation via centralized policies, while NEAT offered focused support through shared workflows, pooled analytics, and direct site engagement, aiding domain team development aligned with local priorities.

### Navigation services sub-stream

The Navigation Services sub-stream was a cornerstone of the IDeA-CTR N3C consortium’s approach to enhancing researcher engagement and data utilization. During the peak two-year period of N3C activity, two project coordinators, providing a combined total of 1.6 full-time equivalent effort, were consistently available to support all participating CTR sites. The sub-stream assisted new members with onboarding, training, communication, and utilization of N3C resources. The sub-stream assisted established members by monitoring and disseminating the frequent evolutionary changes to N3C policy and tools.

Outside this centralized project management team, individual site navigators worked with local investigators to access the consortium resources and navigate the administrative requirements for access. These navigators offered tailored support to address investigators’ specific needs and questions, enhancing the overall research experience within the N3C Enclave. Their efforts ensure that researchers can efficiently utilize the available data, tools, and analytical capabilities, fostering a conducive environment for high-quality research. Each IDeA-CTR had a designated navigator to support investigator needs.

To best guide researchers, this sub-stream created a project proposal form (Supplemental Appendix 1) to help identify the key data points needed for a sound research study, given the large amount of data available in the N3C Enclave. Through these navigation activities, the NEAT workstream provided practical guidance toward the consortium’s goal of advancing the understanding of COVID-19 by facilitating robust and impactful research projects.

### Investigator engagement sub-stream

The Investigator Engagement sub-stream, led by consortium project managers and site navigators, was a key resource that extended the consortium’s reach and enhanced its community engagement. This sub-stream helped recruit new investigators from across the consortium and facilitated their integration into the N3C Enclave and IDeA-CTR consortium. A key component of this sub-stream was hosting investigator-engagement events, which introduced potential new investigators to the resources and opportunities available within the consortium. These events enhanced recruitment, disseminated information about the consortium’s findings, and facilitated the exchange of ideas among researchers. Additionally, the consortium’s presence at IDeA-CTR regional events enabled further expansion of the consortium’s reach within the greater IDeA community.

### Education and training sub-stream

Education and training emerged as key requirements for equipping IDeA-CTR researchers and analysts with the skills necessary to engage in real-world data science. Consequently, a dedicated training and education sub-stream developed comprehensive training modules, workshops, and webinars focusing on data management, analysis, and research methodologies. This initiative reduced the barriers to participation in N3C from an end-user perspective.

The Education and Training sub-stream successfully launched short courses (with two conducted to date, including more than 40 trainees) to equip researchers with the knowledge and skills necessary to utilize the N3C Enclave. These courses addressed technical and analytical skills, reducing barriers for new investigators and improving research quality. Besides hosting these short courses, this sub-stream collaborated with the larger N3C community in the creation and editorial process of *The Researcher’s Guide to N3C* [51]. This guide serves as a living resource for investigators and analysts, offering insights into navigating the N3C Enclave, understanding its data structures, and conducting research effectively.

By co-leading these educational efforts as part of the N3C Education and Training Domain Team (co-founded by the IDeA-CTRs), the consortium underscored its commitment to fostering a well-informed and capable research community. Through the education and training sub-stream, the NEAT workstream facilitated continuous learning and development among consortium members, ensuring they were well-equipped for the evolving challenges of COVID-19 research and beyond.

### Shared analyst pool

Creating a shared analyst pool represents a pivotal innovation within the NEAT workstream, as it addresses the crucial need for analytical support across the consortium’s diverse research projects and institutions with varying analyst availability and skill. The consortium had diverse skills, but no single site could support every project requirement. Pooling resources across the consortium enabled the efficient allocation of analytical expertise, ensuring that investigators–particularly those from sites with limited resources (e.g., lack of biostatistical or health informatics expertise)–could access the necessary support for data analysis within the N3C Enclave. The shared pool consisted of skilled analysts capable of actively curating and analyzing data. We maintained shared logic (definitions, concept sets, analytic pipelines) for replication in similar projects, minimizing work repetition across the consortium. These resources were shared internally at the consortium’s NEAT meetings and externally through the N3C Knowledge Store. While formal tracking of use was not performed, these resources were openly available to the broader N3C community to support research, including rural health observational studies aligned with IDeA-CTR priorities.

Sharing analysts across sites boosted research productivity, efficiency, and quality. Sites lacking dedicated analysts can utilize the consortium’s pool and receive specialized project support from the centralized project management to coordinate the creation of a study team. This collaborative ecosystem not only facilitates the execution of complex analyses but also fosters a culture of shared learning and expertise development within the consortium. By centralizing analytical support, the consortium ensured that research projects benefit from high-quality data analysis, regardless of the individual site’s capacity. This strategy enhanced the consortium’s overall research output, driving meaningful insights into COVID-19 outcomes and contributing to the consortium’s goal of representing the research interests of all IDeA-CTR members.

## Lasting impact of the consortium on collaboration, informatics capacity, and prioritization of IDeA state interests

In addition to advancing data transmission and data use for all IDeA-CTR sites in N3C, the consortium has had a lasting impact on collaboration, capacity development, and research relevant to IDeA states’ health priorities. Site navigators were surveyed in February 2024 regarding their research participation before and after N3C (the survey instrument is available in Supplemental Table 2), receiving an 85% response rate (11 of 13 navigators). Among the participating sites, willingness to participate in research networks was directly impacted by participation in N3C (Table 2). For example, among IDeA-CTR consortium participants, 10 (91%) sites were likely or highly likely to participate in research networks after participating in N3C. Specifically, 10 (91%) sites indicated they would be willing to participate in similar networks (e.g., Observational Health Data Sciences and Informatics [OHDSI] [52] and All of Us) [53] across the IDeA-CTR consortium, while eight (73%) indicated that the consortium increased collaboration with other IDeA-CTR networks. Moreover, 10 (91%) sites indicated that the N3C IDeA-CTR consortium enhanced their clinical capacity. Collectively, the consortium increased collaboration with other IDeA-CTR networks and developed local practice-based research sharing RWD.

**Table 2.** Impact of IDeA-CTR consortium on capacity and collaboration

How likely are you to participate in these research networks now that you’ve participated in N3C?	
Strongly unlikely	0 Sites (0%)
Unlikely	0 Sites (0%)
Neither likely nor unlikely	1 Sites (9.1%)
Likely	4 Sites (36%)
Strongly likely	6 Sites (55%)
Would your site likely participate in other networks similar to N3C across the IDeA-CTR Consortium (e.g., OHDSI, All of Us, IDeA-CTR network)?	
Strongly unlikely	0 Sites (0%)
Unlikely	0 Sites (0%)
Neither likely nor unlikely	1 Sites (9.1%)
Likely	5 Sites (45%)
Strongly likely	5 Sites (45%)
Participating in N3C with the IDeA-CTR Consortium has enhanced the clinical informatics capacity within our network	
Strongly disagree	0 Sites (0%)
Disagree	0 Sites (0%)
Neither agree nor disagree	1 Sites (9.1%)
Agree	4 Sites (36%)
Strongly Agree	6 Sites (55%)
Participation in N3C with the IDeA-CTR Consortium has directly enhanced collaboration with at least one other IDeA-CTR network	
Strongly disagree	0 Sites (0%)
Disagree	0 Sites (0%)
Neither agree nor disagree	3 Sites (27%)
Agree	2 Sites (18%)
Strongly Agree	6 Sites (55%)

## Impact on collaborative efforts

The collaborative efforts within N3C, mainly through the involvement of domain teams, have significantly accelerated the consortium’s research capabilities. Domain teams, spanning 33 clinical areas, have fostered a multidisciplinary approach to COVID-19 research, with leadership roles held in eight of these teams by IDeA-CTR investigators. This structure has enabled the effective coordination of research efforts and the sharing of insights across various specialties, enhancing the consortium’s ability to address complex research questions.

**Figure 4.**
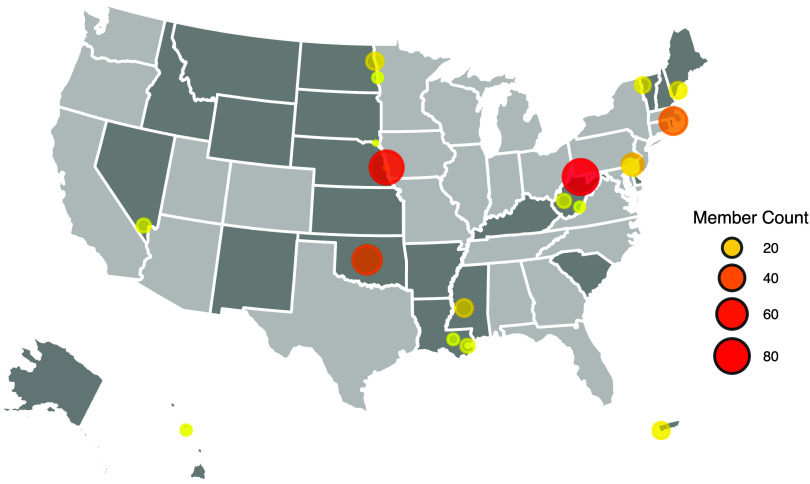
IDeA-CTR N3C consortium collaboration map. shows network collaboration among the IDeA-CTR N3C consortium. IDeA states are **dark grey**, and non-IDeA states are l**ight grey**. This map contains all IDeA-CTR-affiliated sites (*N* = 23) with members participating in N3C (*N* = 401). Each bubble’s size and color intensity represent the relative number of members at each site, with larger and more intensely colored bubbles indicating more participants.

The navigator survey results highlight the positive impact of the consortium’s collaborative framework on participant engagement and willingness to contribute to future research endeavors. Additionally, the consortium’s impact is evident by the number of members from diverse IDeA-CTR-affiliated sites. Figure 4 shows that the IDeA-CTR consortium created a collaborative ecosystem of 401 investigators from 23 IDeA-CTR affiliated sites (based on active N3C DUAs and active users in 2023). Based on the effective collaborative relationships developed through the IDeA-CTR N3C consortium, 11 sites affiliated with 9 IDeA-CTRs formed the IDeA State Consortium for Clinical Research (ISCORE) to conduct clinical trials and observational studies. Two CTSA hubs (the University of Kentucky and the University of Kansas) joined ISCORE. The 13 sites in 10 states and Puerto Rico were one of 15 networks the NIH chose to conduct the Researching COVID to Enhance Recovery (RECOVER) adult observational cohort [54].

## Impact on informatics capacity

The IDeA-CTR consortium has had a lasting impact on regional and national informatics capacity, extending beyond the immediate benefits of COVID-19 research. This impact has improved informatics infrastructure, investigative skills, and collaborative relationships, all of which are crucial for supporting clinical and translational research. Through N3C participation, IDeA-CTRs have enhanced their ability to manage, analyze, and interpret complex health data sets, which has gone far beyond facilitating a more effective response to the pandemic and equipped researchers and analysts within the IDeA-CTRs with advanced informatics tools and capacity, setting a new standard for research excellence within these networks.

Moreover, the collaborative environment fostered by N3C has enriched the IDeA-CTRs’ research ecosystem, enabling the sharing of best practices, data, and resources across a national consortium. This shared learning environment has catalyzed the development of a more cohesive and capable research community adept at addressing complex health challenges more efficiently. In addition to meeting immediate research needs, the centralized analyst and project management pool facilitated longer-term capacity building by enabling participating sites to develop local personnel skilled in RWD analysis.

Locally, the enhancement in informatics capacity has empowered IDeA-CTRs to leverage their capabilities to support their specific research agendas. One specific example is the Southern Center for Maternal Health Equity initiative to pool EHR data across Louisiana, Mississippi, and Arkansas to measure the impact of various interventions, programs, and policies on maternal health and outcomes. Similarly, the Hawai“i Clinical Research Network for Health Equity aims to pool EHR data from two of the largest healthcare systems in Hawai‘i that provide primary care to medically underserved communities. Their efforts included developing and enhancing local practice-based research networks predicated on sharing EHR data. These local enhancements contribute to the national research landscape and strengthen the networks’ roles within their communities.

## Impact on IDeA state health priorities

The IDeA program includes the Commonwealth of Puerto Rico and 23 states, encompassing a diverse range of research interests reflecting the specific needs of these communities. The unique landscape of IDeA states, characterized by rural expanses and diverse underserved populations, presents distinct challenges that necessitate tailored research and healthcare strategies and underscore the complex interplay between socioeconomic factors and health outcomes in these regions. The Rural Health Domain Team, one of several led by IDeA-CTR investigators, is a direct response to the unique challenges experienced by rural populations during the COVID-19 pandemic.

**Figure 5.**
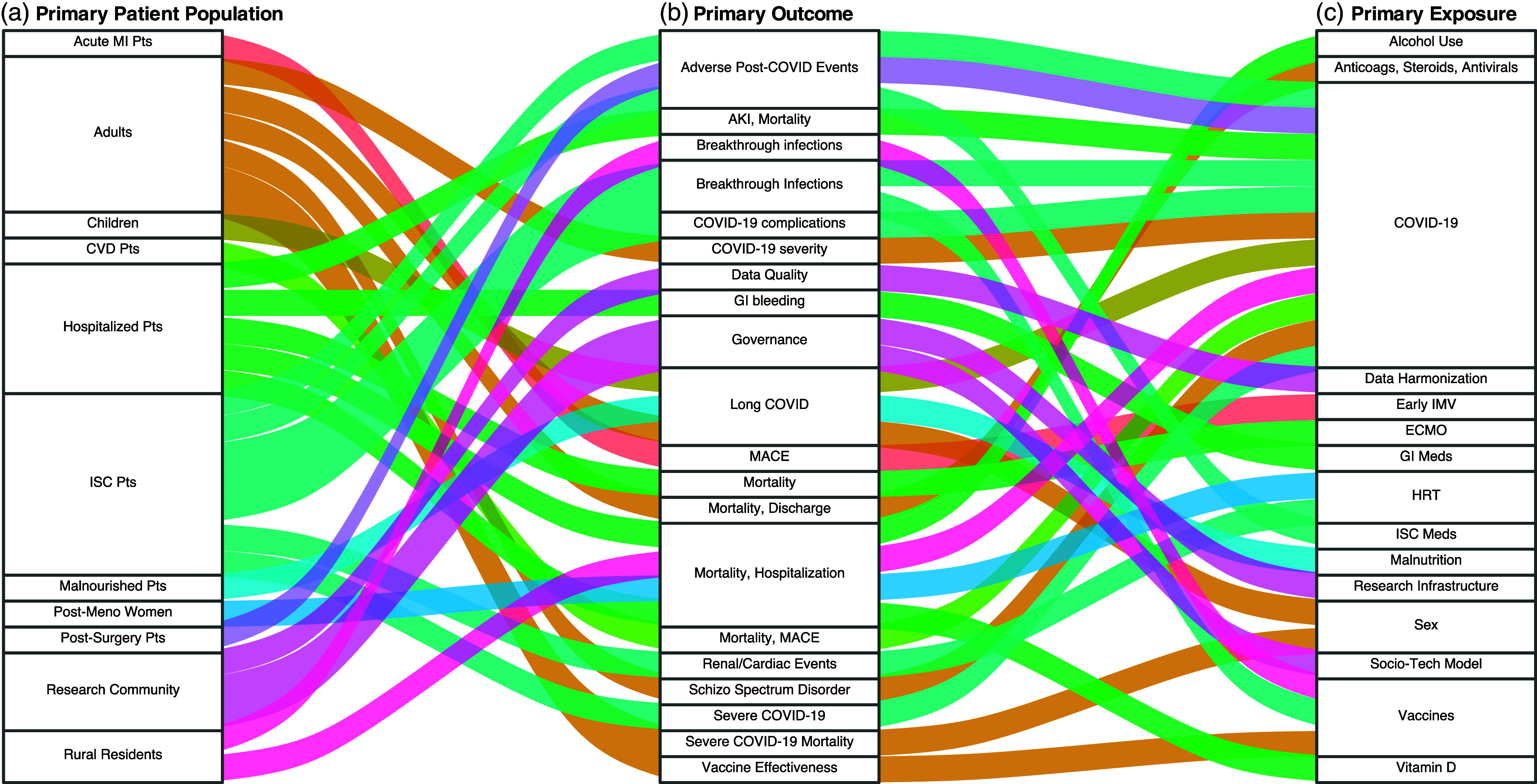
Scope of patient populations, outcomes, and exposures in 29 IDeA-CTR N3C consortium studies, published 2021–2023. shows the scope of 29 IDeA-CTR N3C consortium studies, showcasing some of the priorities addressed in studies published from 2021–2023. The left axis represents A) key patient populations, such as rural residents and immunocompromised patients. The middle axis highlights B) major outcomes, including mortality and breakthrough infections, while the right axis shows C) primary exposures like SARS-CoV-2 infection and vaccination. The plot illustrates how these studies address health disparities and key challenges in IDeA states. The studies and thematic review included in this alluvial plot are available in supplemental Table 3.

Efforts within IDeA-CTRs to address these health priorities are grounded in a deep understanding of the local context, leveraging community engagement and multidisciplinary approaches to develop effective interventions. This localized focus ensures that research initiatives are aligned with the specific health needs of communities, facilitating targeted and impactful health improvements. IDeA-CTR consortium using the N3C Enclave has resulted in 29 manuscripts and 96 presentations at academic and professional conferences from 2021 through 2023, many of which reflect the diverse research interests of IDeA states (Figure 5, Supplemental Table 3).

Moreover, the diversity of research interests within IDeA states highlights the importance of flexible and responsive research infrastructures that, while shared, can still accommodate a broad spectrum of health issues. By prioritizing a range of health concerns that mirror the unique needs of each IDeA state, the IDeA-CTR network has demonstrated the ability to effectively address the multifaceted health challenges facing these diverse regions.

## Discussion

Most research on Team Science and collaborative analytics at scale has focused on the necessary technical architecture [55,56], including data standards and harmonization [57], secure data sharing and access [8], and scalable tools and infrastructure [58]. However, less work has been done regarding the organizational structure required for successful collaboration within clinical and translational research endeavors. Governance structures and communication protocols are crucial in establishing trust, mutual understanding, and shared goals among participating entities [59]. Moreover, applying project management techniques and fostering a collaborative culture [60] are integral to collaborative research’s successful, dynamic, and inclusive nature.

This work presents a flourishing socio-technical ecosystem deployed in response to a global pandemic within the U.S. research ecosystem. The IDeA-CTR N3C consortium’s organizational structure has demonstrated significant advancements in collaborative research for COVID-19, emphasizing shared governance, data management, and educational initiatives. The CTR ecosystem has successfully managed data integration, analysis, and researcher engagement challenges, offering valuable insights into managing and utilizing large-scale health data. By fostering an environment of shared resources and expertise, the consortium enhanced the quality and impact of research within its consortium. The findings highlight the potential for extending this approach to broader real-world data-based research networks and facilitating clinical trials, as is currently being accomplished by the ISCORE clinical trials network.

## Extending the socio-technical architecture to additional research networks

The consortium’s socio-technical ecosystem presents a scalable and adaptable roadmap for enhancing observational research networks like OHDSI, All of Us, or the forthcoming disease-agnostic version of N3C, the National Clinical Cohort Collaborative. The collaborative framework encourages cross-disciplinary research, enables early-stage investigator participation, promotes innovation, and facilitates rapid responses to emerging health challenges. Integrating diverse EHR data and rigorous data quality and management practices could significantly enrich these networks, yielding broader, more nuanced datasets for observational studies. By adopting the consortium’s organizational structure, these networks could leverage enhanced data curation and analysis capabilities, fostering a deeper understanding of various health conditions beyond COVID-19. Moreover, the approach pioneered by the IDeA-CTR N3C consortium does not depend upon centralized databases; it can also be adapted to highly standardized, secure federated research networks, such as machine learning-powered swarm learning [61].

## Leveraging IDeA-CTR consortium for expansion of clinical research

ISCORE was established in 2021, capitalizing on the operational efficiencies, educational scaffolding, and trust developed by the IDeA-CTR consortium’s N3C work. ISCORE is a leading enroller in the RECOVER adult cohort study, and ISCORE’s 13 sites are conducting a clinical trial (NCT05524532) [62] assessing the impact of immulina (an immunomodulator) on inflammatory markers among participants with long COVID. Clinical trial operations, including regulatory oversight, audits for regulatory compliance, and assessment of site performance, are conducted at a lead ISCORE site, providing efficient management. It is anticipated that ISCORE will engage in a broad range of future clinical studies, bringing therapeutic interventions and preventive measures to IDeA state populations who have historically been underrepresented in clinical trials.

The IDeA-CTR N3C consortium’s socio-technical structure can be a scalable framework for future collaborative research, leveraging shared resources to advance scientific discovery and improve health outcomes. The productivity of the IDeA-CTR consortium outperformed what would have been possible if sites had participated separately, demonstrating the potential for extending this model to other research endeavors.

## Conclusions

The IDeA-CTR N3C consortium’s socio-technical ecosystem underscores a transformative approach to team science and shared analysis in health research. By fostering a culture of collaboration among sites from across the U.S., shared governance, and resource pooling, the consortium significantly advanced the efficiency and effectiveness of our COVID-19 research capacity. This collaborative ecosystem set a precedent and provides a roadmap for future collaborative initiatives in health science across IDeA states.

## Supporting information

10.1017/cts.2025.10098.sm001 Anzalone et al. supplementary material Anzalone et al. supplementary material

## Supplementary material

The supplementary material for this article can be found at https://doi.org/10.1017/cts.2025.10098.

## N3C Attribution

The analyses described in this manuscript were conducted with data or tools accessed through the NCATS N3C Data Enclave https://covid.cd2h.org and N3C Attribution & Publication Policy v 1.2-2020-08-25b supported by NCATS Contract No. 75N95023D00001, Axle Informatics Subcontract: NCATS-P00438-B. This research was possible because of the patients whose information is included within the data and the organizations (https://ncats.nih.gov/n3c/resources/data-contribution/data-transfer-agreement-signatories) and scientists who have contributed to the on-going development of this community resource [https://doi.org/10.1093/jamia/ocaa196].

## Data Partners with Released Data

The following institutions whose data is released or pending:

Available: Advocate Health Care Network — UL1TR002389: The Institute for Translational Medicine (ITM) • Aurora Health Care Inc. — UL1TR002373: Wisconsin Network For Health Research • Boston University Medical Campus — UL1TR001430: Boston University Clinical and Translational Science Institute • Brown University — U54GM115677: Advance Clinical Translational Research (Advance-CTR) • Carilion Clinic — UL1TR003015: iTHRIV Integrated Translational health Research Institute of Virginia • Case Western Reserve University — UL1TR002548: The Clinical & Translational Science Collaborative of Cleveland (CTSC) • Charleston Area Medical Center — U54GM104942: West Virginia Clinical and Translational Science Institute (WVCTSI) • Children’s Hospital Colorado — UL1TR002535: Colorado Clinical and Translational Sciences Institute • Columbia University Irving Medical Center — UL1TR001873: Irving Institute for Clinical and Translational Research • Dartmouth College — None (Voluntary) Duke University — UL1TR002553: Duke Clinical and Translational Science Institute • George Washington Children’s Research Institute — UL1TR001876: Clinical and Translational Science Institute at Children’s National (CTSA-CN) • George Washington University — UL1TR001876: Clinical and Translational Science Institute at Children’s National (CTSA-CN) • Harvard Medical School — UL1TR002541: Harvard Catalyst • Indiana University School of Medicine — UL1TR002529: Indiana Clinical and Translational Science Institute • Johns Hopkins University — UL1TR003098: Johns Hopkins Institute for Clinical and Translational Research • Louisiana Public Health Institute — None (Voluntary) • Loyola Medicine — Loyola University Medical Center • Loyola University Medical Center — UL1TR002389: The Institute for Translational Medicine (ITM) • Maine Medical Center — U54GM115516: Northern New England Clinical & Translational Research (NNE-CTR) Network • Mary Hitchcock Memorial Hospital & Dartmouth Hitchcock Clinic — None (Voluntary) • Massachusetts General Brigham — UL1TR002541: Harvard Catalyst • Mayo Clinic Rochester — UL1TR002377: Mayo Clinic Center for Clinical and Translational Science (CCaTS) • Medical University of South Carolina — UL1TR001450: South Carolina Clinical & Translational Research Institute (SCTR) • MITRE Corporation — None (Voluntary) • Montefiore Medical Center — UL1TR002556: Institute for Clinical and Translational Research at Einstein and Montefiore • Nemours — U54GM104941: Delaware CTR ACCEL Program • NorthShore University HealthSystem — UL1TR002389: The Institute for Translational Medicine (ITM) • Northwestern University at Chicago — UL1TR001422: Northwestern University Clinical and Translational Science Institute (NUCATS) • OCHIN — INV-018455: Bill and Melinda Gates Foundation grant to Sage Bionetworks • Oregon Health & Science University — UL1TR002369: Oregon Clinical and Translational Research Institute • Penn State Health Milton S. Hershey Medical Center — UL1TR002014: Penn State Clinical and Translational Science Institute • Rush University Medical Center — UL1TR002389: The Institute for Translational Medicine (ITM) • Rutgers, The State University of New Jersey — UL1TR003017: New Jersey Alliance for Clinical and Translational Science • Stony Brook University — U24TR002306 • The Alliance at the University of Puerto Rico, Medical Sciences Campus — U54GM133807: Hispanic Alliance for Clinical and Translational Research (The Alliance) • The Ohio State University — UL1TR002733: Center for Clinical and Translational Science • The State University of New York at Buffalo — UL1TR001412: Clinical and Translational Science Institute • The University of Chicago — UL1TR002389: The Institute for Translational Medicine (ITM) • The University of Iowa — UL1TR002537: Institute for Clinical and Translational Science • The University of Miami Leonard M. Miller School of Medicine — UL1TR002736: University of Miami Clinical and Translational Science Institute • The University of Michigan at Ann Arbor — UL1TR002240: Michigan Institute for Clinical and Health Research • The University of Texas Health Science Center at Houston — UL1TR003167: Center for Clinical and Translational Sciences (CCTS) • The University of Texas Medical Branch at Galveston — UL1TR001439: The Institute for Translational Sciences • The University of Utah — UL1TR002538: Uhealth Center for Clinical and Translational Science • Tufts Medical Center — UL1TR002544: Tufts Clinical and Translational Science Institute • Tulane University — UL1TR003096: Center for Clinical and Translational Science • The Queens Medical Center — None (Voluntary) • University Medical Center New Orleans — U54GM104940: Louisiana Clinical and Translational Science (LA CaTS) Center • University of Alabama at Birmingham — UL1TR003096: Center for Clinical and Translational Science • University of Arkansas for Medical Sciences — UL1TR003107: UAMS Translational Research Institute • University of Cincinnati — UL1TR001425: Center for Clinical and Translational Science and Training • University of Colorado Denver, Anschutz Medical Campus — UL1TR002535: Colorado Clinical and Translational Sciences Institute • University of Illinois at Chicago — UL1TR002003: UIC Center for Clinical and Translational Science • University of Kansas Medical Center — UL1TR002366: Frontiers: University of Kansas Clinical and Translational Science Institute • University of Kentucky — UL1TR001998: UK Center for Clinical and Translational Science • University of Massachusetts Medical School Worcester — UL1TR001453: The UMass Center for Clinical and Translational Science (UMCCTS) • University Medical Center of Southern Nevada — None (voluntary) • University of Minnesota — UL1TR002494: Clinical and Translational Science Institute • University of Mississippi Medical Center — U54GM115428: Mississippi Center for Clinical and Translational Research (CCTR) • University of Nebraska Medical Center — U54GM115458: Great Plains IDeA-Clinical & Translational Research • University of North Carolina at Chapel Hill — UL1TR002489: North Carolina Translational and Clinical Science Institute • University of Oklahoma Health Sciences Center — U54GM104938: Oklahoma Clinical and Translational Science Institute (OCTSI) • University of Pittsburgh — UL1TR001857: The Clinical and Translational Science Institute (CTSI) • University of Pennsylvania — UL1TR001878: Institute for Translational Medicine and Therapeutics • University of Rochester — UL1TR002001: UR Clinical & Translational Science Institute • University of Southern California — UL1TR001855: The Southern California Clinical and Translational Science Institute (SC CTSI) • University of Vermont — U54GM115516: Northern New England Clinical & Translational Research (NNE-CTR) Network • University of Virginia — UL1TR003015: iTHRIV Integrated Translational health Research Institute of Virginia • University of Washington — UL1TR002319: Institute of Translational Health Sciences • University of Wisconsin-Madison — UL1TR002373: UW Institute for Clinical and Translational Research • Vanderbilt University Medical Center — UL1TR002243: Vanderbilt Institute for Clinical and Translational Research • Virginia Commonwealth University — UL1TR002649: C. Kenneth and Dianne Wright Center for Clinical and Translational Research • Wake Forest University Health Sciences — UL1TR001420: Wake Forest Clinical and Translational Science Institute • Washington University in St Louis — UL1TR002345: Institute of Clinical and Translational Sciences • Weill Medical College of Cornell University — UL1TR002384: Weill Cornell Medicine Clinical and Translational Science Center • West Virginia University — U54GM104942: West Virginia Clinical and Translational Science Institute (WVCTSI) • Submitted: Icahn School of Medicine at Mount Sinai — UL1TR001433: ConduITS Institute for Translational Sciences • The University of Texas Health Science Center at Tyler — UL1TR003167: Center for Clinical and Translational Sciences (CCTS) • University of California, Davis — UL1TR001860: UCDavis Health Clinical and Translational Science Center • University of California, Irvine — UL1TR001414: The UC Irvine Institute for Clinical and Translational Science (ICTS) • University of California, Los Angeles — UL1TR001881: UCLA Clinical Translational Science Institute • University of California, San Diego — UL1TR001442: Altman Clinical and Translational Research Institute • University of California, San Francisco — UL1TR001872: UCSF Clinical and Translational Science Institute NYU Langone Health Clinical Science Core, Data Resource Core, and PASC Biorepository Core — OTA-21-015A: Post-Acute Sequelae of SARS-CoV-2 Infection Initiative (RECOVER) Pending: Arkansas Children’s Hospital — UL1TR003107: UAMS Translational Research Institute • Baylor College of Medicine — None (Voluntary) • Children’s Hospital of Philadelphia — UL1TR001878: Institute for Translational Medicine and Therapeutics • Cincinnati Children’s Hospital Medical Center — UL1TR001425: Center for Clinical and Translational Science and Training • Emory University — UL1TR002378: Georgia Clinical and Translational Science Alliance • HonorHealth — None (Voluntary) • Loyola University Chicago — UL1TR002389: The Institute for Translational Medicine (ITM) • Medical College of Wisconsin — UL1TR001436: Clinical and Translational Science Institute of Southeast Wisconsin • MedStar Health Research Institute — None (Voluntary) • Georgetown University — UL1TR001409: The Georgetown-Howard Universities Center for Clinical and Translational Science (GHUCCTS) • MetroHealth — None (Voluntary) • Montana State University — U54GM115371: American Indian/Alaska Native CTR • NYU Langone Medical Center — UL1TR001445: Langone Health’s Clinical and Translational Science Institute • Ochsner Medical Center — U54GM104940: Louisiana Clinical and Translational Science (LA CaTS) Center • Regenstrief Institute — UL1TR002529: Indiana Clinical and Translational Science Institute • Sanford Research — None (Voluntary) • Stanford University — UL1TR003142: Spectrum: The Stanford Center for Clinical and Translational Research and Education • The Rockefeller University — UL1TR001866: Center for Clinical and Translational Science • The Scripps Research Institute — UL1TR002550: Scripps Research Translational Institute • University of Florida — UL1TR001427: UF Clinical and Translational Science Institute • University of New Mexico Health Sciences Center — UL1TR001449: University of New Mexico Clinical and Translational Science Center • University of Texas Health Science Center at San Antonio — UL1TR002645: Institute for Integration of Medicine and Science • Yale New Haven Hospital — UL1TR001863: Yale Center for Clinical Investigation.
