# Supplementary material for: Building a collaborative ecosystem across the IDeA-CTR networks in response to a public health emergency
Source: J Clin Transl Sci. 2025 Jul 16;9(1):e168. doi: 10.1017/cts.2025.10098 (PMC12444709; doi:10.1017/cts.2025.10098)
Supplement: Anzalone et al. supplementary material [file S2059866125100988sup001.docx]

**Supplementary Online Content**

*Building a Collaborative Ecosystem Across the IDeA-CTR Networks in Response to a Public Health Emergency*

**Table of Contents**

1. ***Appendix A.*** *National COVID Cohort Collaborative (N3C) Project Proposal Form and Agreement*
2. ***Supplemental Tables:***

***Supplemental Table 1.*** *Quarterly Reporting Metrics*

***Supplemental Table 2.*** *Data Collection Instrument Distributed to N3C IDeA-CTR Consortium Navigators*

***Supplemental Table 3.*** *IDeA-CTR N3C Consortium Manuscripts and Thematic Overview of Study Population, Outcomes, and Exposures, 2021-2023*

1. ***Supplemental References***

**Appendix A. National COVID Cohort Collaborative (N3C) Project Proposal Form and Agreement**

**Project Title:**

**CTR Site:**

**CTR N3C Navigator:**

**Data Level:**

**If Associated with Domain Team, list Domain Team:**

**Project Lead(s):**

Name and contact information

**Study team: (SME, BERD member, etc.):**

**Study Design, including statistical or data science requirements:**

**Will this project need an ML/AI analyst?**

**Prediction analysis (ML/DL-decision making)?**

**Will a hypothesis be tested?**

**Summary of the project:**

**Research Question:**

**Patient Population(s)/Cohorts (inclusion/exclusion criteria)Study Endpoints:**

- Primary
- Secondary

**Data Elements Needed (please list for each category): Please include relevant, specific medical codes (e.g., ICD-10-CM, LOINC, SNOMED CT, CPT) where possible**

- Baseline Patient Characteristics:
  - Include baseline inclusion/exclusion criteria
  - Treatment Group
  - Control Group
  - Time frame
- Pre-existing conditions and timing of those conditions **– SNOMED CT or ICD-10-CM, if possible** (prior to COVID hospital admission/past medical history, during hospitalization or active COVID infection, following discharge, etc.)
- Discharge diagnosis(es):
- Laboratory- **LOINC codes, if possible** (please include item and timing of lab value or other criteria (e.g. mean/median, minimum, maximum, on admission, etc.))
  - Brand name and generic
  - Dosages and frequency
- Medications and timing of use – **RxNorm or NDC, if possible** (e.g. prior to index hospitalization admission, during index hospitalization, after index hospital discharge)
  - Generic name
  - Dosages, frequency, duration, form, route if needed
  - ingredient(s)
- Interventions and procedures - **SNOMED CT, CPT4, if possible** (e.g. mechanical ventilation, extracorporeal membrane oxygenation, surgery(ies), percutaneous coronary intervention, etc.).
- Primary and Secondary Outcome(s) (e.g., death, discharge disposition, mechanical ventilation, ECMO, vasopressor use, etc.)
- Other measurements (e.g., BMI, BP)

Reviewed by CTR N3C Analyst: ___________ (name and date)

Approved by CTR N3C Analyst: ___________ (name and date)

Approved by site N3C PI: ___________ (name and date)

| **Action:** | **Date Completed:** | **Notes:** |
| --- | --- | --- |
| Analyst has access to project |  |  |
| Analyst has IRB approval |  |  |
| Workspace has been provisioned with in the enclave |  |  |
| Meeting date and time has been established: At minimum to meet weekly for 1 hour |  |  |
| Investigator provides analyst with DUR |  |  |
| Calendar invites with zoom link have been sent (navigator) |  |  |
| Statistical Review completed |  |  |
| Statistician has access to project |  |  |
| Timeline of deliverables has been established |  |  |
| Timeline of deliverables has been created |  |  |
| Concept sets have been reviewed and approved |  |  |
| Study definitions and cohort criteria have been reviewed and approved |  |  |
| Writing of manuscript expectations have been agreed upon |  | Suggestions: content expert writes the intro, background, interpretation/discussion  Analyst: write the methods, study design, cohort selection |
| Review of code and logic with another analyst |  | Periodic review is ideal |
| WVCTSI has entered project into iLab |  |  |
| Data Download request submitted to N3C |  |  |
| Publication Request submitted to N3C |  |  |
| Submitted abstract: |  |  |

**Supplemental Table 1. Quarterly Reporting Metrics**

| **Site:** | **Site 1** | **Site N** |
| --- | --- | --- |
| Number of Engaged Investigators |  |  |
| Number of Data Use Requests (DURS) submitted by Site Investigators |  |  |
| Number of DURS in which Site Investigators participating |  |  |
| Number of Abstracts-Submitted |  |  |
| Number of Presentations |  |  |
| Number of Manuscripts Published |  |  |
| Number of Investigator Engagement Events attended by >1 site investigator |  |  |
| Number of EHR Payloads entered in N3C |  |  |
| Number of Domain teams in which site investigators participate |  |  |
| Number of Domain teams led by site investigators |  |  |
| Funding proposal submitted based on N3C |  |  |
| Number of SARS-CoV-2 sequences submitted |  |  |

**Supplemental Table 2. Data Collection Instrument Distributed to N3C IDeA-CTR Consortium Navigators**

| **Question 1. Did you participate in any research networks before N3C (e.g., OHDSI, PCORnet, ACT, i2b2, TriNetX)?**  Options:   1. Yes 2. No |
| --- |
| **Question 1.A. If yes, which network?**  Text response. |
| **Question 1.B. If yes, did you participate in funded studies in these networks?**  Options:   1. Yes 2. No |
| **Question 2. What is the source data you are submitting from?**   1. Single health system 2. Multiple health systems 3. Health Information Exchange (HIE) 4. Other (please specify): |
| **Question 3. How likely are you to participate in these networks now that you've participated in N3C?**   1. Strongly unlikely 2. Unlikely 3. Neither likely nor unlikely 4. Likely 5. Strongly Likely |
| **Question 4. Please rate the following statement: Participating in N3C with the IDeA-CTR Consortium has enhanced the clinical informatics capacity within our network.**   1. Strongly disagree 2. Disagree 3. Neither agree nor disagree 4. Agree 5. Strongly Agree |
| **Question 5. Participation in N3C with the IDeA-CTR Consortium has directly enhanced collaboration with at least one other IDeA-CTR network.**   1. Strongly disagree 2. Disagree 3. Neither agree nor disagree 4. Agree 5. Strongly Agree |
| **Question 3. Would your site likely participate in other networks similar to N3C across the IDeA-CTR Consortium (e.g., OHDSI, All of Us, IDeA-CTR network)?**   1. Strongly unlikely 2. Unlikely 3. Neither likely nor unlikely 4. Likely 5. Strongly Likely |

**Supplemental Table 3. IDeA-CTR N3C Consortium Manuscripts and Thematic Overview of Study Population, Outcomes, and Exposures, 2021-2023**

| **Study Title** | **Population** | **Outcome** | **Exposure** |
| --- | --- | --- | --- |
| The relationship between body mass index and mortality is not linear in patients requiring venovenous extracorporeal support^1^ | Patients hospitalized with COVID-19 | Mortality | ECMO |
| SARS-CoV-2 Infection is Associated with an Increase in New Diagnoses of Schizophrenia Spectrum and Psychotic Disorder: A Study Using the US National COVID Cohort Collaborative (N3C)^2^ | Adults | Schizophrenia Spectrum and Psychotic Disorder (SSPD) | SARS-CoV-2 infection |
| The N3C governance ecosystem: A model socio-technical partnership for the future of collaborative analytics at scale^3^ | Research community | Governance | Socio-Technical Model |
| Outcomes of patients with active cancers and pre-existing cardiovascular diseases infected with SARS-CoV-2^4^ | Cancer, cardiovascular disease | Major Adverse Cardiovascular Events (MACE), Mortality | SARS-CoV-2 infection |
| Effectiveness of various COVID-19 vaccine regimens among 10.4 million patients from the National COVID Cohort Collaborative during Pre-Delta to Omicron periods – United States, 11 December 2020 to 30 June 2022^5^ | Adults | Vaccine effectiveness | COVID-19 vaccines |
| Associations between COVID-19 therapies and inpatient gastrointestinal bleeding: A multisite retrospective study^6^ | Patients hospitalized with COVID-19 | Gastrointestinal bleeding | GI Medications |
| Thirty-Day Mortality and Complication Rates in Total Joint Arthroplasty After a Recent COVID-19 Diagnosis: A Retrospective Cohort in the National COVID Cohort Collaborative (N3C)^7^ | Post-surgery patients | Mortality, Pneumonia, Sepsis, Myocardial Infarction | SARS-CoV-2 infection |
| A machine learning-based phenotype for long COVID in children: An HER-based study from the RECOVER program^8^ | Pediatric patients | Long COVID | SARS-CoV-2 infection |
| Geographic and Temporal Trends in COVID-Associated Acute Kidney Injury in the National COVID Cohort Collaborative^9^ | Patients hospitalized with COVID-19 | Acute Kidney Injury (AKI), Mortality | SARS-CoV-2 infection |
| Hormone replacement therapy and COVID-19 outcomes in solid organ transplant recipients compared with the general population^10^ | Immunosuppressed/compromised patients | Mortality, Renal/cardiac events | Hormone Replacement Therapy (HRT) |
| Effect of menopausal hormone therapy on COVID-19 severe outcomes in women – A population-based study of the US National COVID Cohort Collaborative (N3C) data^11^ | Post-menopausal women | Mortality, Hospitalization | Hormone Replacement Therapy (HRT) |
| Assessing the effects of therapeutic combinations on SARS-CoV-2 infected patient outcomes: A big data approach^12^ | Adults | Mortality, Discharge | Anticoagulants, Steroids, Antivirals |
| Community risks for SARS-CoV-2 infection among fully vaccinated US adults by rurality: A retrospective cohort study from the National COVID Cohort Collaborative^13^ | Rural residents | Breakthrough infections | COVID-19 vaccines |
| Higher hospitalization and mortality rates among SARS-CoV-2-infected persons in rural America^14^ | Rural residents | Hospitalization, Mortality | SARS-CoV-2 infection |
| Impact of malnutrition on clinical outcomes in patients diagnosed with COVID-19^15^ | Malnourished patients | Post-acute sequelae of COVID-19 | Malnutrition |
| Sex differences in determinants of COVID-19 severe outcomes – findings from the National COVID Cohort Collaborative (N3C)^16^ | Adults | Severe COVID-19 outcomes, Mortality | Sex |
| The impact of COVID-19 on clinical outcomes among acute myocardial infarction patients undergoing early invasive treatment strategy^17^ | Acute Myocardial Infarction patients | Mortality, Major adverse cardiac events (MACE) | Early invasive treatment |
| An ordinal severity scale for COVID-19 retrospective studies using Electronic Health Record data^18^ | Adults | COVID-19 severity scale | SARS-CoV-2 infection |
| The risk and consequences of breakthrough SARS-CoV-2 infection in solid organ transplant recipients relative to non-immunosuppressed controls^19^ | Immunosuppressed/compromised patients | Breakthrough infections, Mortality | SARS-CoV-2 infection |
| Association of Vitamin D Prescribing and Clinical Outcomes in Adults Hospitalized with COVID-19^20^ | Hospitalized | Mortality, Hospitalization | Vitamin D prescription |
| COVID-19 patients with documented alcohol use disorder or alcohol-related complications are more likely to be hospitalized and have higher all-cause mortality^21^ | Hospitalized | Mortality, Hospitalization | Alcohol use disorder |
| Synergies between centralized and federated approaches to data quality: a report from the national COVID cohort collaborative^22^ | Research community | Data quality improvement | Data harmonization |
| Association Between Immune Dysfunction and COVID-19 Breakthrough Infection After SARS-CoV-2 Vaccination in the US^23^ | Immunosuppressed/compromised patients | Breakthrough infections | COVID-19 vaccines |
| Long-term use of immunosuppressive medicines and in-hospital COVID-19 outcomes: a retrospective cohort study using data from the National COVID Cohort Collaborative^24^ | Immunosuppressed/compromised patients | Mortality, Inpatient Adverse Events | Immunosuppressive medication |
| Sex and organ-specific risk of major adverse renal or cardiac events in solid organ transplant recipients with COVID-19^25^ | Immunosuppressed/compromised patients | Major Adverse Renal or Cardiac Events (MARCE), Mortality | SARS-CoV-2 infection |
| Characterizing Long COVID: Deep Phenotype of a Complex Condition^26^ | Adults | Long COVID | Sex |
| COVID-19 in Solid Organ Transplantation: Results of the National COVID Cohort Collaborative^27^ | Immunosuppressed/compromised patients | Mortality, COVID-19 complications | SARS-CoV-2 infection |
| COVID-19 Disease Severity among People with HIV Infection or Solid Organ Transplant in the United States: A Nationally-representative, Multicenter, Observational Cohort Study^28^ | Immunosuppressed/compromised patients | Severe COVID-19 outcomes | SARS-CoV-2 infection |
| The National COVID Cohort Collaborative (N3C): Rationale, design, infrastructure, and deployment^29^ | Research community | Governance | Research Infrastructure |

**Supplemental References**

1. Tham E, Campbell S, Hayanga H, et al. The relationship between body mass index and mortality is not linear in patients requiring venovenous extracorporeal support. *J Thorac Cardiovasc Surg*. Oct 2024;168(4):1107-1115. doi:10.1016/j.jtcvs.2023.11.041

2. Rahman A, Russell M, Zheng W, Eckrich D, Ahmed I. SARS-CoV-2 Infection is Associated with an Increase in New Diagnoses of Schizophrenia Spectrum and Psychotic Disorder: A Study Using the US National COVID Cohort Collaborative (N3C). *medRxiv*. Dec 5 2023;doi:10.1101/2023.12.05.23299473

3. Suver C, Harper J, Loomba J, et al. The N3C governance ecosystem: A model socio-technical partnership for the future of collaborative analytics at scale. *J Clin Transl Sci*. 2023;7(1):e252. doi:10.1017/cts.2023.681

4. Patel B, Chapman SA, Neumann JT, et al. Outcomes of patients with active cancers and pre-existing cardiovascular diseases infected with SARS-CoV-2. *Cardiooncology*. Oct 6 2023;9(1):36. doi:10.1186/s40959-023-00187-w

5. Fu Y, Wu K, Wang Z, et al. Effectiveness of various COVID-19 vaccine regimens among 10.4 million patients from the National COVID Cohort Collaborative during Pre-Delta to Omicron periods - United States, 11 December 2020 to 30 June 2022. *Vaccine*. Oct 6 2023;41(42):6339-6349. doi:10.1016/j.vaccine.2023.08.069

6. Wiedel NA, Sayles H, Larson J, et al. Associations between COVID-19 therapies and inpatient gastrointestinal bleeding: A multisite retrospective study. *J Med Virol*. Oct 2023;95(10):e29100. doi:10.1002/jmv.29100

7. Pincavitch JD, Pisquiy JJ, Wen S, et al. Thirty-Day Mortality and Complication Rates in Total Joint Arthroplasty After a Recent COVID-19 Diagnosis: A Retrospective Cohort in the National COVID Cohort Collaborative (N3C). *J Bone Joint Surg Am*. Sep 6 2023;105(17):1362-1372. doi:10.2106/jbjs.22.01317

8. Lorman V, Razzaghi H, Song X, et al. A machine learning-based phenotype for long COVID in children: An EHR-based study from the RECOVER program. *PLoS One*. 2023;18(8):e0289774. doi:10.1371/journal.pone.0289774

9. Yoo YJ, Wilkins KJ, Alakwaa F, et al. Geographic and Temporal Trends in COVID-Associated Acute Kidney Injury in the National COVID Cohort Collaborative. *Clin J Am Soc Nephrol*. Aug 1 2023;18(8):1006-1018. doi:10.2215/cjn.0000000000000192

10. Vinson AJ, Anzalone A, Schissel M, et al. Hormone replacement therapy and COVID-19 outcomes in solid organ transplant recipients compared with the general population. *Am J Transplant*. Apr 26 2023;doi:10.1016/j.ajt.2023.04.020

11. Yoshida Y, Chu S, Zu Y, Fox S, Mauvais-Jarvis F. Effect of menopausal hormone therapy on COVID-19 severe outcomes in women - A population-based study of the US National COVID Cohort Collaborative (N3C) data. *Maturitas*. Apr 2023;170:39-41. doi:10.1016/j.maturitas.2022.10.005

12. Moradi H, Bunnell HT, Price BS, et al. Assessing the effects of therapeutic combinations on SARS-CoV-2 infected patient outcomes: A big data approach. *PLoS One*. 2023;18(3):e0282587. doi:10.1371/journal.pone.0282587

13. Anzalone AJ, Sun J, Vinson AJ, et al. Community risks for SARS-CoV-2 infection among fully vaccinated US adults by rurality: A retrospective cohort study from the National COVID Cohort Collaborative. *PLoS One*. 2023;18(1):e0279968. doi:10.1371/journal.pone.0279968

14. Anzalone AJ, Horswell R, Hendricks BM, et al. Higher hospitalization and mortality rates among SARS-CoV-2-infected persons in rural America. *J Rural Health*. Jun 27 2022;doi:10.1111/jrh.12689

15. Ponce J, Anzalone AJ, Bailey K, et al. Impact of malnutrition on clinical outcomes in patients diagnosed with COVID-19. *JPEN J Parenter Enteral Nutr*. Nov 2022;46(8):1797-1807. doi:10.1002/jpen.2418

16. Yoshida Y, Chu S, Fox S, et al. Sex differences in determinants of COVID-19 severe outcomes - findings from the National COVID Cohort Collaborative (N3C). *BMC Infect Dis*. Oct 12 2022;22(1):784. doi:10.1186/s12879-022-07776-7

17. Sharma P, Shah K, Loomba J, et al. The impact of COVID-19 on clinical outcomes among acute myocardial infarction patients undergoing early invasive treatment strategy. *Clin Cardiol*. Oct 2022;45(10):1070-1078. doi:10.1002/clc.23908

18. Khodaverdi M, Price BS, Porterfield JZ, et al. An ordinal severity scale for COVID-19 retrospective studies using Electronic Health Record data. *JAMIA Open*. Oct 2022;5(3):ooac066. doi:10.1093/jamiaopen/ooac066

19. Vinson AJ, Anzalone AJ, Sun J, et al. The risk and consequences of breakthrough SARS-CoV-2 infection in solid organ transplant recipients relative to non-immunosuppressed controls. *Am J Transplant*. Oct 2022;22(10):2418-2432. doi:10.1111/ajt.17117

20. Fairfield KM, Murray KA, Anzalone AJ, et al. Association of Vitamin D Prescribing and Clinical Outcomes in Adults Hospitalized with COVID-19. *Nutrients*. Jul 26 2022;14(15)doi:10.3390/nu14153073

21. Bailey KL, Sayles H, Campbell J, et al. COVID-19 patients with documented alcohol use disorder or alcohol-related complications are more likely to be hospitalized and have higher all-cause mortality. *Alcohol Clin Exp Res*. Jun 2022;46(6):1023-1035. doi:10.1111/acer.14838

22. Pfaff ER, Girvin AT, Gabriel DL, et al. Synergies between Centralized and Federated Approaches to Data Quality: A Report from the National COVID Cohort Collaborative. *J Am Med Inform Assoc*. Sep 30 2021;doi:10.1093/jamia/ocab217

23. Sun J, Zheng Q, Madhira V, et al. Association Between Immune Dysfunction and COVID-19 Breakthrough Infection After SARS-CoV-2 Vaccination in the US. *JAMA Intern Med*. Dec 28 2021;doi:10.1001/jamainternmed.2021.7024

24. Andersen KM, Bates BA, Rashidi ES, et al. Long-term use of immunosuppressive medicines and in-hospital COVID-19 outcomes: a retrospective cohort study using data from the National COVID Cohort Collaborative. *Lancet Rheumatol*. Jan 2022;4(1):e33-e41. doi:10.1016/s2665-9913(21)00325-8

25. Vinson AJ, Dai R, Agarwal G, et al. Sex and organ-specific risk of major adverse renal or cardiac events in solid organ transplant recipients with COVID-19. *Am J Transplant*. Jan 2022;22(1):245-259. doi:10.1111/ajt.16865

26. Deer RR, Rock MA, Vasilevsky N, et al. Characterizing Long COVID: Deep Phenotype of a Complex Condition. *EBioMedicine*. Dec 2021;74:103722. doi:10.1016/j.ebiom.2021.103722

27. Vinson AJ, Agarwal G, Dai R, et al. COVID-19 in Solid Organ Transplantation: Results of the National COVID Cohort Collaborative. *Transplant Direct*. Nov 2021;7(11):e775. doi:10.1097/txd.0000000000001234

28. Sun J, Patel RC, Zheng Q, et al. COVID-19 Disease Severity among People with HIV Infection or Solid Organ Transplant in the United States: A Nationally-representative, Multicenter, Observational Cohort Study. *medRxiv*. Jul 28 2021;doi:10.1101/2021.07.26.21261028

29. Haendel MA, Chute CG, Bennett TD, et al. The National COVID Cohort Collaborative (N3C): Rationale, design, infrastructure, and deployment. *J Am Med Inform Assoc*. Mar 1 2021;28(3):427-443. doi:10.1093/jamia/ocaa196
